# Nitric Oxide Inactivation Mechanisms in the Brain: Role in Bioenergetics and Neurodegeneration

**DOI:** 10.1155/2012/391914

**Published:** 2012-06-10

**Authors:** Ricardo M. Santos, Cátia F. Lourenço, Ana Ledo, Rui M. Barbosa, João Laranjinha

**Affiliations:** Faculty of Pharmacy and Center for Neurosciences and Cell Biology, University of Coimbra, Health Sciences Campus, Azinhaga de Santa Comba, 3000-548 Coimbra, Portugal

## Abstract

During the last decades nitric oxide (^•^NO) has emerged as a critical physiological signaling molecule in mammalian tissues, notably in the brain. ^•^NO may modify the activity of regulatory proteins via direct reaction with the heme moiety, or indirectly, via S-nitrosylation of thiol groups or nitration of tyrosine residues. However, a conceptual understanding of how ^•^NO bioactivity is carried out in biological systems is hampered by the lack of knowledge on its dynamics *in vivo*. Key questions still lacking concrete and definitive answers include those related with quantitative issues of its concentration dynamics and diffusion, summarized in the how much, how long, and how far trilogy. For instance, a major problem is the lack of knowledge of what constitutes a physiological ^•^NO concentration and what constitutes a pathological one and how is ^•^NO concentration regulated. The ambient ^•^NO concentration reflects the balance between the rate of synthesis and the rate of breakdown. Much has been learnt about the mechanism of ^•^NO synthesis, but the inactivation pathways of ^•^NO has been almost completely ignored. We have recently addressed these issues *in vivo* on basis of microelectrode technology that allows a fine-tuned spatial and temporal measurement ^•^NO concentration dynamics in the brain.

## 1. Introduction

Nitric oxide (^•^NO) is a small and diffusible free radical synthesized by a family of nitric oxide synthases (NOS) that participates in a wide range of signaling pathways in biological tissues, mediating physiologic processes such as vasodilation, memory and learning, neuronal development, regulation of immune response, among many others [1–5]. In the brain, ^•^NO is mainly synthesized in synaptic terminals by a neuronal NOS isoform, acting as a neuromodulator [3, 4]. The radical nature, small size, and hydrophobicity support the notion that ^•^NO lacks specific interactions with receptors, and yet these properties confer to this molecule a great versatility concerning interactions with biological targets. The outcome of these interactions is dictated by ^•^NO concentration dynamics, ranging from physiological to pathological effects leading to cell death. This dual role anticipates a tight regulation of ^•^NO concentration dynamics under physiologic conditions.

The major aspects that characterize ^•^NO neuroactivity and its regulation are discussed in this paper.

## 2. The Interactions of ^•^NO with Biological Targets: Redox and Functional Impact

Nitric oxide is able to rapidly diffuse in tissue and interact with a variety of biological targets involved in relevant physiological processes. Two main mechanisms that stabilize the unpaired electron of ^•^NO are its reaction with other free radicals and interactions with d-orbitals of transition metals [6]. Among the latter, the interactions of ^•^NO with iron are the most relevant in biological systems due to the abundance of proteins containing iron, most notably hemeproteins, involved in numerous physiological processes. Essentially, ^•^NO can interact with iron complexes by three ways: (a) binding to iron, (b) reaction with dioxygen iron complexes, and (c) reaction with high valent oxo-complexes [7]. ^•^NO can bind to both ferrous and ferric heme proteins, but the binding to Fe(II) is generally faster and more reversible than to Fe(III) [8]. Actually, the majority of biologically relevant ^•^NO reactions with heme proteins involve the reversible binding of ^•^NO to ferrous iron in proteins, a process called nitrosylation [7]. For instance, the binding of ^•^NO to ferrous heme activates the enzyme soluble guanylyl cyclase (sGC) [3], which is the best characterized signaling target of ^•^NO and inhibits cytochrome c oxidase (CcO), a crucial enzyme for mitochondrial respiration [9]. The interaction between ^•^NO and ferrous hemoglobin is also biologically relevant by binding to the deoxy-hemoglobin heme or as a means to degrade ^•^NO via reaction with oxy-hemoglobin, resulting in the oxidation of the ferrous heme and formation of nitrate.

These interactions are relatively fast, exhibiting rate constants of 2–4 × 10^7^ M^−1^ s^−1^ [8]. Given the abundance of hemoglobin in the vasculature, they constitute the main pathway of ^•^NO removal in that compartment and significantly contribute to shape the dynamics of ^•^NO at the neighboring tissues [10].

A critical aspect of the interactions of ^•^NO with hemeproteins able to transduce a transient ^•^NO concentration change into a biological response is their different sensitivity for ^•^NO. The most sensitive ^•^NO physiologic signaling target is sGC with half-maximal activation at 10 nM [11], mediating most of the known ^•^NO biological effects [3]. For higher ^•^NO concentrations inhibition of CcO occurs, being half-maximal at *≈*120 nM, under resting metabolic conditions and physiological O_2_ tension [9, 12].

The indirect effects of ^•^NO require it to react with molecular oxygen or superoxide anion radical (O_2_^−•^) to produce reactive oxygen and nitrogen species (RONS) such as nitrogen dioxide (^•^NO_2_), nitrogen trioxide (N_2_O_3_), and peroxynitrite (ONOO^−^). ONOO^−^ and ^•^NO_2_ are potent oxidants (>1.0 V NHE) [13] and can both oxidize and nitrate protein residues and lipids. The product of ^•^NO autooxidation, N_2_O_3_, is a mild oxidant and will preferably nitrosate nucleophiles such as amines and thiols [14, 15].

S-Nitrosation is a covalent posttranslational modification associated to ^•^NO-dependent signaling, which refers to the incorporation of a nitroso group (–NO) to a thiol group (in cystein residues). There are several mechanisms by which S-nitrosation can occur, mainly through (a) involvement of N_2_O_3_, (b) direct interaction of ^•^NO with a thiyl radical (radical-radical interaction), and (c) transfer of a nitroso group from a nitrosylated metal or nitrosothiol (transnitrosation) [16]. S-Nitrosation mediated by N_2_O_3_ somehow defines the microenvironment in which the modification predominantly occurs. Because the reaction of ^•^NO with O_2_ is slow, it requires high levels of both species, a condition favored by the proximity of sources of ^•^NO production and by hydrophobic environment (where both species accumulate). Also, the life time of N_2_O_3_ is increased in hydrophobic compartments [7]. These factors limit S-nitrosation and confer selectivity to the reaction as only cysteine residues found within a particular microenvironment in a protein are prone to be nitrosated (reviewed by [17]).

Several proteins have been described as being regulated by S-Nitrosation and ensued physiologic and pathologic consequences described [18, 19]. To mention some, S-nitrosation of N-methyl-D-aspartate receptors (NMDAr) has been shown to inactivate the receptor, thereby possibly protecting against excessive NMDAr activation and consequent excitotoxicity [20]. S-Nitrosation of 2-amino-3-(5-methyl-3-oxo-1,2-oxazol-4-yl)propanoic acid receptor (AMPAr) regulatory proteins (stargazin and N-ethylmaleimide-sensitive factor) may increase surface expression of the receptor [21, 22]. Other examples include nitrosation of caspases-3 and -8 and poly(ADP-ribose) polymerase [23–25], protecting against apoptosis. In turn, S-nitrosation of glyceraldehyde-3-phosphate dehydrogenase (GAPDH) inhibits its dehydrogenase activity and induces an acyl phosphatase activity in the enzyme, resulting in the uncoupling of glycolytic flux from ATP synthesis [26]. Nitrosated GAPDH can also translocate to the nucleus, enabling it to degrade selected target proteins and affect apoptosis [27].

The nitration of proteins and lipids by ^•^NO-derived reactive species is a further posttranslational modification of proteins by which ^•^NO can accomplish functional diversity in cellular processes. It is currently accepted that protein nitration is an *in vivo* protein modification that translates into functional alterations in physiological and pathological settings [28]. Nitration results from the addition of a nitro –NO_2_ group to aromatic and aliphatic residues in proteins or to the aliphatic chain of fatty acids, mainly mediated by ONOO^−^ and ^•^NO_2_. In proteins, tyrosine residues are the key target for nitration by ONOO^−^ (reviewed in [29]). 3-Nitrotyrosine (3-NT) has been used as a marker of pathological events associated to oxidative stress. Indeed, 3-NT immunoreactivity has been found in early stages of several neurodegenerative disorders in human autopsy samples as well as in animal models (reviewed in [28, 30]).

Protein nitration is a very selective modification. Not all tyrosine residues present in a given protein can suffer nitration. Protein folding, the surrounding local environment (namely, the presence of glutamate residues), and the nitration agent all contribute to direct nitration towards specific tyrosine residues [31]. Examples of nitration targets with relevance in the context of the nervous system and neurodegeneration may include the following: (1) neurofilament L in human ALS neurons, preventing assembly and possibly axonal transport, both pathological hallmarks of ALS [32–34]; (2) tyrosine hydroxylase, the rate-limiting enzyme in catecholamine synthesis, causing loss of enzyme function in the MPTP-induced Parkinson's disease model [35–37], affording a possible cause of dopamine deficiency prior to cell death; (3) nitration of Mn superoxide dismutase inactivates this mitochondrial antioxidant enzyme, with implications several disease states (reviewed in [38]); (4) Lewy bodies in Parkinson's disease have been found to contain nitrated alfa-synuclein, which tends to form oligomers [39].

In sum, at low ^•^NO concentrations, direct ^•^NO interactions with transition metal centers via reversible binding are likely to predominate as a physiological signal transduction mechanism.

On the other hand, the actual concentration at which ^•^NO-dependent nitrosative and nitrative chemistry occurs, either from a physiological or deleterious view point, is not known and likely depends on the particular tissue redox conditions. A rough estimate has been made in cellular preparations, indicating that nitrosative stress becomes significant when the ^•^NO concentration reaches near-micromolar levels [5, 40].

From the lines of evidence shown above it becomes clear that the concentration dynamics of ^•^NO (the profile of change in time and space) is an essential aspect of its biology. Nitric oxide signals, having different amplitude, time course, and spatial distribution may thus encode different signaling messages, possibly mediated by different biological targets. Paradoxically, in spite of being one of the most studied endogenous molecules during the last decades, the exceeding majority of studies are of qualitative and phenomenological nature, lacking the critical quantitative information on ^•^NO dynamics. Under this scenario it is not a surprise that several dogmas have undermined the knowledge of ^•^NO biological actions.

Therefore, the monitoring of ^•^NO concentration profiles *in vivo*, allowing to unravel its endogenous concentration profiles and the major factors regulating its concentration, is a critical piece of knowledge to understand the mechanisms by which ^•^NO affects cell and tissue function in the brain.

## 3. The Transient ^•^NO Change from the Background and the Mechanisms That Regulate Its Profile

### 3.1. ^•^NO Production in the Brain

The ^•^NO signaling in CNS is intimately associated to the glutamate system. In glutamatergic synapses, ^•^NO synthesis involves the stimulation of ionotropic glutamate receptors (iGluR), particularly NMDA subtype, and consequent influx of Ca^2+^ to the cytosol that, upon binding to calmodulin, activates neuronal isoform of NOS (nNOS). The *α*-splice variant of nNOS is particular in that it possesses an N-terminal PDZ motif [41], which allows the enzyme to bind to other PDZ-containing proteins, such as the synaptic density scaffold protein PSD-95 [42]. The functional impact of this association is very relevant, as PSD-95 simultaneously binds to the NR2 subunit of the NMDAr [43, 44], thus forming a supramolecular complex that places the Ca^2+^-dependent nNOS under the direct effect of Ca^2+^ influx through the activated NMDAr channel [45]. Upon Ca^2+^ influx, providing that substrates (L-arginine, O_2_) and several other cofactors (NADPH, FMN, FAD, tetrahydrobiopterin, heme) are available, nNOS catalyses the conversion of L-arginine to L-citrulline and ^•^NO [46, 47]. However, the regulation of ^•^NO synthesis by nNOS goes beyond Ca^2+^ dynamics, also involving, among other factors, specific adaptor proteins [48] and posttranslational modifications [49, 50]. In addition to the diverse regulatory mechanisms associated to ^•^NO synthesis, the distribution of nNOS within a particular volume is suggested to influence the ^•^NO volume signaling [51]. In essence, the abovementioned notions suggest an intricate regulatory process for ^•^NO production that may translate into distinct concentration dynamics.

#### 3.1.1. Measurement of Nitric Oxide Concentration Dynamics *In Vitro* and *In Vivo*

The measurement of ^•^NO in real time by electrochemical methods with microelectrodes inserted into the ^•^NO diffusion field is an important tool to characterize ^•^NO concentration dynamics [52]. Glutamate-dependent ^•^NO production in hippocampus is of particular relevance because of the involvement of ^•^NO in the regulation of plasticity, such as learning and memory, and cell death associated with neurodegeneration [53]. Electrochemical detection presents several advantages in relation to other methods to measure ^•^NO in biological systems, as it allows direct and real-time measurement. Moreover, the small size of the carbon fiber microelectrodes affords their use in nervous tissue, with minimal perturbation of the natural environment, and confers high spatial resolution to the measurements [54, 55].

In hippocampal slices, chemically modified carbon fiber microelectrodes with suitable analytical properties for ^•^NO-selective measurement [56] have been used to perform real-time recording of endogenous ^•^NO concentration dynamics evoked by activation of ionotropic GluR. By using this methodological approach we were able to show that NMDA-evoked ^•^NO concentration dynamics is heterogeneous along the trisynaptic loop in the rat hippocampus [57]. We also provided evidence that the AMPAr in addition to the NMDAr could contribute to the fine tuning of glutamate-dependent ^•^NO production [58] and that NMDA-evoked ^•^NO production inhibits tissue O_2_ consumption for submicromolar concentrations [59].

The use of this approach has also allowed the characterization of endogenous ^•^NO concentration dynamics produced in rat brain *in vivo* upon glutamatergic stimulation. In the hippocampus we found that both endogenous and synthetic agonists of ionotropic GluR (glutamate, NMDA, and AMPA) promoted transient increases of extracellular ^•^NO concentration, although with different kinetics. Pharmacological modulation suggested that ^•^NO overflow elicited by glutamate resulted from an integrated activation of both subtypes of ionotropic GluR [60]. Glutamate-evoked ^•^NO concentration changes were further characterized along the hippocampal trisynaptic loop (CA1, CA3, and dentate gyrus), as well as in cerebral cortex and striatum, showing that while glutamate induced transitory increases in ^•^NO levels in all regions, regional-specific concentration profiles were observed (unpublished data).

### 3.2. ^•^NO Diffusion and Half-Life

Together with ^•^NO production, the rates of diffusion and inactivation are key determinants of ^•^NO concentration dynamics in tissues. However, unlike the mechanisms of ^•^NO synthesis, the physiologic processes underlying the termination of ^•^NO signals *in vivo* (inactivation) remain unclear, particularly in the brain. Inactivation of classical neurotransmitters (e.g., dopamine or glutamate) relies on their rapid removal from the extracellular space via intracellular uptake processes [61–63]. However, given the uncommon physicochemical properties of ^•^NO, its consumption via chemical reactions is generally accepted as the most probable route of inactivation. Although several biochemical mechanisms have been proposed to fulfill that role, the exact extent of their effect on ^•^NO concentration dynamics in brain tissue has remained uncertain. Another layer of complexity is added by the effect of diffusional processes on the signal. Although ^•^NO has been frequently considered as a molecule that can freely diffuse in tissues [64–66], with a diffusion coefficient estimated in aqueous solution of 2 × 10^−5^ to 4.5 × 10^−5^ cm^2^/s [67–69], it has been observed that ^•^NO diffusion is hindered across the aortic wall [70].

Recently, we have obtained experimental data that allowed the characterization of ^•^NO diffusion in the rat brain *in vivo*. The studies were conducted by using ^•^NO-selective carbon fiber microelectrodes to monitor ^•^NO increases following local application of small volumes (few nanoliters) of ^•^NO solution from a micropipette located 200–350 *μ*m away. These microelectrode/micropipette arrays were stereotaxically inserted in the brain of anesthetized rats [71].

The resulting electrochemical signals were fitted to an equation that describes the diffusion of a compound and takes into account a first-order kinetics of inactivation. This approach allowed the estimation of the ^•^NO diffusion coefficient and half-life in tissue. We found that ^•^NO is highly diffusible and short lived in the brain, having an effective diffusion coefficient (D_NO_*) of 2.  2 × 10^−5^ cm^2^/s and a half-life of 0.64 s in the rat cortex.

We have also investigated possible pathways of ^•^NO diffusion by testing the concepts of free, hindered, and enhanced diffusion. The D_NO_ obtained in agarose gel, a model used to evaluate ^•^NO-free diffusion, was 2.6 × 10^−5^ cm^2^/s, only 14% higher than the *in vivo *D_NO_*, suggesting that ^•^NO could freely diffuse in the brain. Accordingly, we found that ^•^NO diffusion in brain tissue is distinct from a molecule of similar size that remains in the extracellular space (nitrite), but importantly, our data indicated that ^•^NO diffusion is hindered by conditions that mimic intracellular macromolecular crowding [71].

These latter lines of evidence suggest that neither ^•^NO diffusion through the extracellular space nor a homogeneous diffusion in the tissue through brain cells provides a reasonable conceptual explanation for the similarity between the D_NO_* obtained *in vivo* and the D_NO_ found in agarose gel. Thus, as previously suggested for O_2_ [72, 73], it is likely that ^•^NO diffusion in nervous tissue is facilitated by certain physiological processes. A likely candidate is ^•^NO partition in hydrophobic media, such as cell membranes and myelin sheaths, which may constitute low-resistance pathways that facilitate ^•^NO diffusion in nervous tissue, resulting in an increased diffusion rate.

Together with diffusion, the quantification of ^•^NO half-life in the brain is also important to better understand the dynamics of ^•^NO, produced during brain function. ^•^NO half-life is a kinetic parameter essential for the definition of its basal concentrations and diffusion radius. Some studies have estimated the ^•^NO half-life in biological tissues in the range between 5 and 15 s [66]. Studies performed on isolated cell preparations of brain and liver extrapolated values for *in vivo* of around 100 ms [74, 75], in accordance with the half-life obtained in heart muscle [76]. In intact brain tissue, a half-life of 10 ms was reported in acute cerebellar slices for [^•^NO] below 10 nM [77] which was 60 fold slower in organotypic cerebellar slices [78]. Although variability exists in the data, these works support the notion that ^•^NO is a short-lived messenger, in agreement with the biological necessity to maintain ^•^NO levels within the physiological range. Our *in vivo* approach, based on the fitting of a diffusion/inactivation equation to the signals of exogenously applied ^•^NO *in vivo* in the rat brain cortex, allowed the estimation of a half-life of 0.64 s in that brain region, thus providing quantitative experimental evidence for a sub-second ^•^NO half-life *in vivo*.

### 3.3. Mechanisms of ^•^NO Inactivation

Several studies have found O_2_-dependent mechanisms of ^•^NO consumption/inactivation in *in vitro* preparations but the direct reaction between ^•^NO and O_2_ is too slow to account for significant ^•^NO consumption if one considers the low concentration of the reactants *in vivo* (particularly ^•^NO). Nevertheless, the favored partition of these molecules in the hydrophobic phase of cell membranes greatly accelerates the reaction, possibly accounting for ^•^NO consumption in tissues [79].

As mentioned above, ^•^NO direct biological activity may play out through the reaction with transition metals, in particular the iron contained in hemeproteins. One such target for ^•^NO is Cytochrome c oxidase (CcO), the terminal complex of the mitochondrial respiratory chain. In the mid-1990s, ^•^NO was shown to bind to and inhibit CcO [80] and block mitochondrial respiration in preparations as diverse as isolated mitochondria [81] synaptosomes [82], and primary cell cultures [83, 84].

The initial assessment of ^•^NO interaction with CcO suggested an inhibition mechanism based on the high-affinity, reversible binding of ^•^NO to the enzyme's binuclear active site, in competition with O_2_. [81, 82, 85]. In accordance with this model that prevails under high enzyme turnover conditions, ^•^NO binds to the fully reduced binuclear center (heme a_3_/Cu_B_) and its removal from the active binuclear sites returns CcO to a fully active state.

A second low-affinity inhibitory site has been proposed for the fully oxidized enzyme—the Cu_B_ in the binuclear center-rendering CcO inactive [86, 87]. This is an uncompetitive mechanism of inhibition of CcO by ^•^NO. Contrary to the simple on/off mechanism observed in the competitive model, bound ^•^NO reduces the enzyme and is itself oxidized to NO_2_^−^. Enzyme inhibition is reverted by dissociation of NO_2_^−^ upon further reduction [88, 89]. This uncompetitive inhibition mechanism is favored by high [O_2_] and low turnover and its main biological role seems to be to consume ^•^NO, thus shaping ^•^NO concentration dynamics in the tissue [90, 91].

In mitochondria, ^•^NO can also be consumed by the nearly diffusion-limited reaction with O_2_^•−^ [92], generated as a byproduct of cellular respiration. However, the physiologic relevance of this reaction is questionable, since O_2_^•−^ dismutation by MnSOD greatly decreases O_2_^•−^ concentration (in spite of the favoured competition of ^•^NO over SOD to O_2_^•−^) to values in the pM range under physiologic conditions [7, 93].

Several heme-containing enzymes catalyze redox reactions that can consume ^•^NO in dispersed preparations. Among others, a flavoheme protein in several mammalian cell lines [94], lipoxygenases, prostaglandin H synthase and cycloxygenase-1 in platelets [95–97], peroxidases [98], and cytochrome P450 oxidoreductase [99] have been identified.

Proteins from the globin family are other likely candidates to participate in ^•^NO inactivation in brain tissue due to their rapid reaction with ^•^NO (*k* ≈ 10^7^ M^−1^ s^−1^). In several brain regions, neurons express neuroglobin (Ngb), cytoglobin, and also hemoglobin [100–102]. The exact physiologic functions of these proteins in the brain tissue are not clear. It was observed that the overexpression of Ngb protects the brain against ischemic damage [103] and cells overexpressing Ngb are more resistant to ^•^NO cytotoxicity, suggesting a possible role of ^•^NO scavenging in the neuroprotective mechanism of Ngb. However, given the low intraneuronal concentration of these proteins (<1 *μ*M), the function of Ngb as a robust ^•^NO scavenger would require its association with a putative met-Ngb reductase to rapidly regenerate ferrous heme upon reaction with ^•^NO. Yet, this functional association has not been reported [104]. Neuronal hemoglobin appears to have a role in intracellular oxygen storage or transport but insufficient data exists regarding its capacity to metabolize ^•^NO in neurons [102]. Conversely, it is widely accepted that hemoglobin is the major ^•^NO sink in the vasculature, due to its high concentration in erythrocytes (ca. 20 mM) and high reaction rate with ^•^NO [105]. The effectiveness of this reaction on the regulation of ^•^NO concentration in the extravascular compartment has been disputed since erythrocytic packing of hemoglobin and intravascular flow can decrease the effectiveness of hemoglobin scavenging of ^•^NO by 3-4 orders of magnitude [105–107].

The uncertainty regarding how ^•^NO is inactivated in the brain in physiologic conditions is related to difficulties in its direct measurement in intact tissue. A study on ^•^NO inactivation in cerebellar slices of rat brain found that ^•^NO was inactivated by an unknown mechanism that could not be explained by any known mechanism of ^•^NO consumption [77].

#### 3.3.1. Pathways of ^•^NO Inactivation *In Vivo*

**Figure 1:**
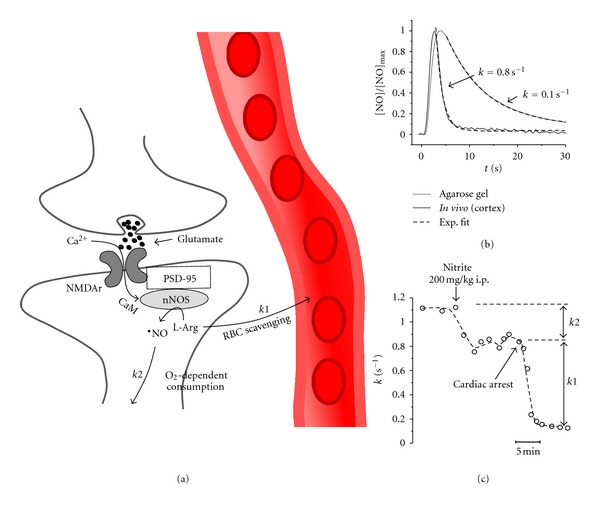
The major pathways of ^•^NO production and inactivation in the brain. (a) ^•^NO is synthesized following calcium entrance into the postsynaptic density (upon glutamate activation of NMDA receptors). Calcium activates nNOS by promoting Calmodulin (CaM) binding to the enzyme. ^•^NO rapidly diffuses to neighboring tissue, being inactivated both by O_2_-dependent mechanisms and by scavenging by circulating erythrocytes (RBCs). (b) Typical electrochemical signals obtained using microelectrodes in the rat brain *in vivo* and in agarose gel following local application of small volumes (few nL) of ^•^NO solution. First-order decay constant values (*k*) were used to quantify the decay profiles. (c) Anoxia, induced by a nitrite lethal dose, induced a 20% decrease in *k* (*k2*), in contrast with a large decrease in *k* following cardiac arrest, suggesting that the major route of ^•^NO inactivation in the brain *in vivo *is by circulating RBCs scavenging (*k1*). Adapted from [10].

A strategy to the study of the mechanisms that govern ^•^NO inactivation *in vivo* has included the recordings of ^•^NO signals by ^•^NO-selective microelectrodes, following local application of small volumes of exogenous ^•^NO in the brain [10]. The decay of ^•^NO signals obtained by means of this approach was very sensitive to experimental conditions impairing vascular function *in vivo*. First, global ischemia induced a 90% decrease in the ^•^NO signals decay rate constant (*k*), suggesting that ^•^NO inactivation is nearly abolished during this condition. Second, the *k* values of ^•^NO signals decay were 3–5-fold higher *in vivo* than in brain slices of cortex and hippocampus, which lack functional vasculature. Finally, impairing the microcirculation in the brain *in vivo* by inducing hemorrhagic shock induced an average 50% decrease in *k*. Comparatively, modulation of O_2_ tension in the brain *in vivo, *either by inducing hypoxia or hyperoxia, caused only small changes in ^•^NO decay (20%), thus demonstrating that scavenging by circulating red blood cells constitutes the major ^•^NO inactivation pathway in the brain (Figure 1).

The ^•^NO half-life in tissue is thereby expected to be dependent on the vascular density, which may be tuned to meet specific local signaling requirements. Accordingly, using microelectrode arrays that allow monitoring ^•^NO in four brain sites simultaneously, we observed that the probability of ^•^NO detection following its local application is apparently related with the local vascular density [10]. 

## 4. The Functional Impact of ^•^NO in Brain Tissue

### 4.1. ^•^NO as a Neuromodulator

sGC is the best characterized signaling target for ^•^NO and is often considered a receptor-like molecule for ^•^NO in cells for the role of ^•^NO as a neuromodulator is mainly mediated by its binding to sGC, leading to transient increases in cGMP.

The most widespread mechanism associated with cGMP is the activation of the cGMP-dependent protein kinase (PKG). Several substrates for PKG have been identified and many of its actions are exerted at the level of phosphatases, thereby affecting the levels of phosphorylation of effector proteins [108]. Other mechanism able to mediate ^•^NO signaling effects downstream sGC is the cGMP activation of cyclic nucleotide-gated (CNG) ion channels [109] and hyperpolarization-activated cyclic nucleotide-modulated (HCN) channels [110]. CNG and HCN are nonselective cation channels that allow the passage of several ions, including Na^+^, K^+^, and Ca^2+^. For instance, studies reported that, in neurons expressing these ionic channels, the ^•^NO-cGMP pathway cause a pre- or postsynaptic membrane depolarization (depending on the location of the channels) thereby modulating neuronal excitability [3].

It is noteworthy that the actions of ^•^NO through the ^•^NO-cGMP pathway obey no general rules. Despite employing the same transduction mechanism (cGMP), the physiological outcomes are tissue/cell specific since different cell populations may arbor different cGMP targets. Thus, the great variability of cGMP targets among different populations of neurons seems to explain the wide range of reported ^•^NO effects in the nervous system. To cite some examples, ^•^NO has been implicated in the modulation of neuronal excitability, synaptic plasticity, modulation of neurotransmitter release, regulation of rhythmic activity, and neurovascular coupling [3, 4, 46, 111].

### 4.2. Regulation of Mitochondrial Respiration

The average [O_2_] in capillaries is 30 *μ*M and typically cells experience an intracellular concentration of O_2_ of around 3 *μ*M [112]. The high affinity of CcO for its substrate guarantees sustained mitochondrial phosphorylation with a large safety margin regarding O_2_ [113]. ^•^NO will compete with O_2_ for binding to the active site of CcO, inhibiting respiration, raising the enzyme's K_m_ and limiting O_2_ usage even under normoxic conditions.

The physiological and/or pathophysiological impact of ^•^NO inhibition of CcO will depend on the fraction of enzyme that is effectively inhibited and if this decreases O_2_ consumption and oxidative phosphorylation [114]. The overall electron flow in the respiratory chain is typically regulated by complex I in state 4 respiring mitochondria and under these conditions, a fractional inhibition of CcO by ^•^NO produces no net change on O_2_ consumption, contrary to what occurs in state 3 respiring mitochondria [115, 116]. An immediate consequence of decreased O_2_ consumption is the increase in O_2_ availability and increased tissue oxygenation at sites farther away from blood vessels [74], an effect that may act in synergy with ^•^NO-induced vasodilatiion. Alternatively, increased [O_2_] may render it available to participate in signaling pathways such as the activation of hypoxia-inducible factor [117].

A key notion which has emerged in the literature is that mitochondrial cell signaling cascades are intimately linked to ^•^NO regulation of CcO function [118]. The interaction of ^•^NO with CcO may inhibit enzyme activity without producing a net effect in cellular respiration: steady-state and kinetic modeling [119] have revealed that the ^•^NO-CcO interaction can lead to an accumulation of reduced cytochromes upstream of CcO without producing a net effect on cellular respiration, but with consequences in redox signaling pathways such as increased mitochondrial production RONS, notably O_2_^−•^ and H_2_O_2_ [114, 120, 121], both of which can impact downstream signaling cascades.

The situation changes dramatically when inhibition of CcO is severe and persistent-excessive inhibition of mitochondrial respiration results in bioenergetics dysfunction and cellular damage, conditions associated with aging and neurodegeneration. Under such conditions, excess O_2_^−•^ may react with ^•^NO yielding ONOO^−^, which unlike ^•^NO can irreversibly block all complexes of the mitochondrial respiratory chain through oxidation and nitration chemistry [122–124]. In 2005, Shiva et al. proposed the term nitroxia to describe this pathological situation resulting from a deregulation of the ^•^NO-CcO signaling pathway with increased production of RONS leading to mitochondrial and cellular oxidation and nitration chemistry [125].

### 4.3. Neurovascular Coupling

^•^NO has been suggested as a mediator of the neurovascular coupling, the active mechanism enlarging vessel diameter in response to the rising of metabolic demands imposed by neuronal activity [126]. Indeed, ^•^NO appears well suited to mediate this process as it is a potent vasodilator, is released during enhanced neuronal activity, and, as previously discussed, is also highly diffusible. During the years, the role of ^•^NO in neurovascular coupling has been strengthened by the observation that the increase in cerebral blood flow dependent on neuronal activation is repressed by NOS inhibitors [127–130]. However, contradictory reports have also shown the lack of effect of NOS inhibitors, pointing towards a lack of ^•^NO-mediated effect associated to neurovascular coupling [131–133]. As a matter of fact, although it is plausible that neuronal-derived ^•^NO is involved in the regulation of cerebral blood flow, concrete evidence is still missing, as well as the elucidation of the underlying mechanism [134]. Recently, by using an experimental approach that allowed the simultaneous measurement of ^•^NO concentration dynamics and cerebral blood flow changes *in vivo* upon glutamatergic activation in hippocampus we were able to establish the temporal, amplitude, and spatial association of both events. Furthermore, the coupling between neuronal activation and local cerebral blood flow changes mediated by neuronal-derived ^•^NO occurs in the hippocampus regardless of the intermediacy of other cellular players such as astrocytes (unpublished data).

The mechanism of neuronal-derived ^•^NO, via volume signaling, mediating neurovascular coupling, may be a noncanonical fashion to underlie a process of vital importance for the brain to preserve its structural and functional integrity [135]. However, the dual interaction of ^•^NO concentration dynamics and vasculature assists the hypothesis of a highly and intrinsically controlled mechanism to match blood supply with the metabolic demands imposed by increased neuronal activity. While ^•^NO triggers the increase in cerebral blood flow, in turn, the increase in the cerebral blood flow, by way of hemoglobin-dependent inactivation of ^•^NO, helps to shape the ^•^NO signal.

### 4.4. Neurodegeneration

A key tenet of ^•^NO bioactivity is that besides participating in important physiological functions as those previously mentioned, it has also been implicated in pathological processes associated with several neurodegenerative disorders, such as Alzheimer's disease (AD), Parkinson's disease (PD), amyotrophic lateral sclerosis (ALS), Huntington's disease (HD), and ischemic brain injury (reviewed by [111, 136]. The pathological role of ^•^NO involves pathways that are only partially different from those underlying its physiological actions but which are associated to other stress conditions. In AD, the three NOS isoforms are suggested to operate as central mediators of amyloid-*β* (A*β*) action, contributing to the maintenance, self-perpetuation, and progression of the disease [137], although data regarding changes in NOS in AD are highly inconsistent [138]. In cell-free assays, A*β* peptides were shown to strongly inhibit constitutive NOS (eNOS and nNOS) [139]. Conversely, other reports have shown A*β*-dependent enhancement of nNOS activity [140], and memory impairment appears to be correlated with the increase in nNOS expression and ^•^NO levels [141].

A larger consensus exists regarding the inducible isoform of NOS, which seems to be overexpressed in AD [138]. Various studies have reported that A*β* stimulates microglial and astrocytic ^•^NO production [142–144]. Moreover, modifications of iNOS expression are suggested to importantly contribute to AD progression. The ablation of iNOS from a transgenic mouse model of AD protected the AD-like mice from cerebral plaque formation and increased A*β* levels, astrocytosis, and microgliosis [145]. Moreover, astrocytic-derived ^•^NO triggers tau hyperphosphorylation in hippocampal neurons [146] and A*β*-mediated inhibition of NMDAr-dependent LTP requires iNOS activity [147].

The majority of neurotoxic effects of ^•^NO supporting the development of AD pathological mechanisms are due to indirect reactions of ^•^NO, promoting posttranslational protein modifications, namely, nitration and S-nitrosation. Indeed, high levels of nitrotyrosine have been found in brains from AD patients [148–151]. Nitrated proteins were described to be associated with A*β* deposition [150], and recently A*β* itself was shown to be a target for ^•^NO bioactivity. Nitrated A*β* is characterized by an accelerated aggregation rate, being detected in the core of plaques of APP/PS1 mice and AD brains [152]. Also tau protein and synaptophysin are potential targets for nitration with relevant consequences in terms of AD progression [153].

Furthermore, lines of evidence suggest that a number of proteins are S-nitrosated in AD. An example with important impact in neurodegeneration is the S-nitrosation of endoplasmic reticulum (ER) chaperone protein-dissulphide isomerase (PDI). The modification of an active cysteine in the PDI promotes the inhibition of both isomerase and chaperone activities, resulting in abnormal accumulation of misfolded and polyubiquitinated proteins, ER stress, and ultimately in cell death [154, 155]. Also dynamin-related protein 1 (Drp1) is a target for S-nitrosation, being hyperactivated, a mechanism suggested to underlie A*β*-related mitochondrial fission and neuronal injury [156].

Finally, it should be remarked that ^•^NO may also have a protective role in the development of AD pathology. Under physiological conditions, endothelium-derived ^•^NO evidenced a protective action against A*β* accumulation through direct modulation of A*β*, APP, and BACE-1 levels [157].

## 5. Novel Perspectives on the Mechanisms Underlying Deregulation of ^•^NO Dynamics in Disease

As mentioned in the sections above, the excessive activation of NOSs is commonly regarded as the main mechanism leading to the buildup of cytotoxic ^•^NO concentrations in pathological conditions ranging from AD, PD, multiple sclerosis (MS) to ischemic damage [158, 159]. Despite the view of the excessive ^•^NO production in neurodegeneration (although the quantitation *in vivo* requires refinenments), the notions presented in the previous sections of this paper also highlight the role of the pathways of ^•^NO diffusion and inactivation in the regulation of ^•^NO levels in brain tissue. Accordingly, a pathological change of the mechanisms controlling diffusion and inactivation might also cause a deregulation in ^•^NO concentration dynamics with consequences for tissue homeostasis. Indeed, given the recently found importance of the vasculature on the regulation of ^•^NO inactivation *in vivo* [10], there are several pathological situations in which a vascular impairment might greatly account for deregulation of ^•^NO levels, including ischemia-reperfusion, AD, and MS.

It is known that, in the brain of AD patients, vascular dysfunction appears in the early stages of the disease, which manifests as a characteristic oligemia in some brain regions [160]. This condition might contribute to increase ^•^NO concentration due to lowered inactivation.

Conversely, the blood brain barrier breakdown that occurs in MS [161] might contribute to increase ^•^NO inactivation, thereby lowering ^•^NO concentration. But, alterations in inactivation might also be beneficial in some circumstances. We observed a great decrease in ^•^NO inactivation rate in the brain during global ischemia [10], which might potentiate ^•^NO accumulation in the affected tissue, formed from either residual NOS activity or NOS-independent ^•^NO synthesis mechanisms, such as ischemic reduction of nitrite [162]. Interestingly, preischemic administration of ^•^NO donors or nitrite *in vivo* decreases brain ischemia/reperfusion infarct volume in models of focal ischemia [162]. The vasodilatory action of ^•^NO may explain its neuroprotective role during ischemia by enhancing microcirculation in the regions adjacent to the affected area (penumbra). Thus, it is possible that the impairment of ^•^NO inactivation in the brain region affected by ischemia is protective by increasing local ^•^NO availability and consequently enhancing microcirculation in the adjacent tissue.
